# System wide channel network analysis reveals hotspots of morphological change in anthropogenically modified regions of the Ganges Delta

**DOI:** 10.1038/s41598-020-69688-3

**Published:** 2020-07-30

**Authors:** Teresa Jarriel, Leo F. Isikdogan, Alan Bovik, Paola Passalacqua

**Affiliations:** 1 Department of Civil, Architectural and Environmental Engineering and Center for Water and the Environment, The University of Texas at Austin, 10100 Burnet Rd., MC R8000, Austin, TX 78758 USA; 2 Department of Electrical and Computer Engineering, The University of Texas at Austin, 2501 Speedway, Austin, TX 78712 USA

**Keywords:** Geomorphology, Hydrology, Environmental impact

## Abstract

The Ganges Brahmaputra Meghna Delta (GBMD) is a large and complex coastal system whose channel network is vulnerable to morphological changes caused by sea level rise, subsidence, anthropogenic modifications, and changes to water and sediment loads. Locating and characterizing change is particularly challenging because of the wide range of forcings acting on the GBMD and because of the large range of scales over which these forcings act. In this study, we examine the spatial variability of change in the GBMD channel network. We quantify the relative magnitudes and directions of change across multiple scales and relate the spatial distribution of change to the spatial distribution of a variety of known system forcings. We quantify how the channelization varies by computing the Channelized Response Variance (CRV) on 30 years of remotely sensed imagery of the entire delta extent. The CRV analysis reveals hotspots of morphological change across the delta. We find that the magnitude of these hotspots are related to the spatial distribution of the dominant physiographic forcings in the system (tidal and fluvial influence levels, channel connectivity, and anthropogenic interference levels). We find that the anthropogenically modified embanked regions have much higher levels of geomorphic change than the adjacent natural Sundarban forest and that this change is primarily due to channel infilling and increased rates of channel migration. Having a better understanding of how anthropogenic changes affect delta channel networks over human timescales will help to inform policy decisions affecting the human and ecological presences on deltas around the world.

## Introduction

Many river deltas are large and complex systems whose channels evolve in response to changes in internal and external forcings. Changes to water inflow, sediment load, land use, subsidence, modern climate, and anthropogenic fluvial modification structures such as dams, levees, embankments, and dredging operations can all play a role in delta morphodynamics. With more than 500 million people living on river deltas around the world^1^, detecting the magnitudes of changes to deltas is critical for managing these systems, and it is especially important to detect the portions of the system that are changing the most. The goal of this study is to examine a large delta channel network to identify if change is occurring, where hotspots of change are occurring, what the rates of change are, and what may be causing the changes in certain areas to be higher than others. This information will help us better understand morphodynamic patterns in delta systems and make predictions to inform policy decisions affecting the human and ecological presences on deltas.

Deltas are currently threatened by a variety of factors. As inflow conditions of water and sediment change, channel movement such as channel widening, narrowing, lateral migrations, meandering, and avulsions all act to rework delta landscapes. In addition to the natural morphological evolution of delta systems, changes can also be anthropogenically forced. Processes like construction of dams and embankments^2–4^, accelerated subsidence^1,5–8^, and sediment mining operations^9–12^, have all been shown to affect the natural resilience of delta systems to sea level rise and salinity intrusion. The anthropogenic disturbances themselves as well as their effects are spatially variable, so determining the locations on a delta where the hotspots of most extreme changes are occurring is necessary for focusing remediation efforts.

**Figure 1:**
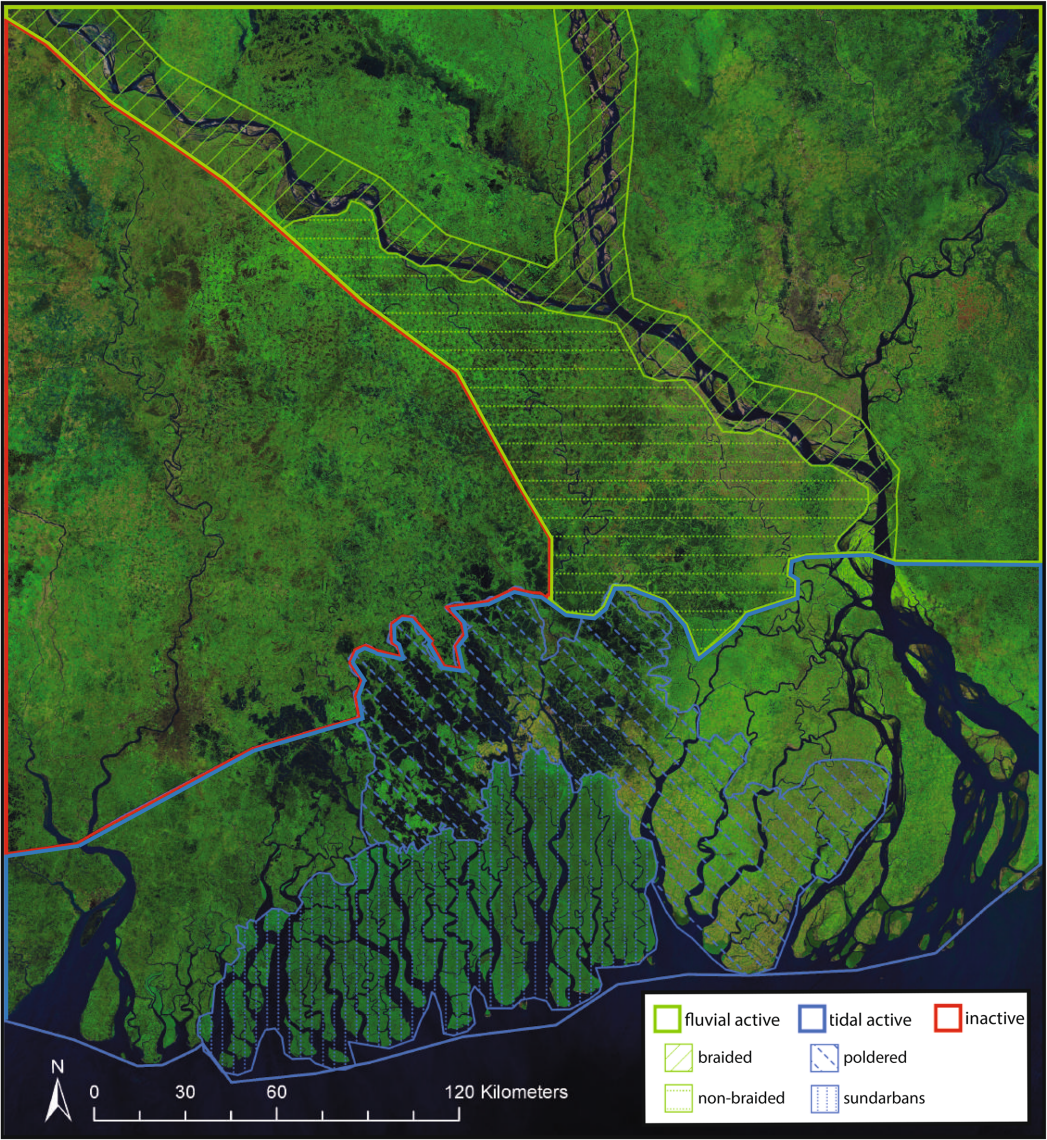
Landsat imagery of the GBMD from 2019 with physiographic zones overlaid^24,36,37^. Landsat imagery created by mosaicking all cloud free images from 10/1/2019 to 3/31/2020. Poldered zones determined from field observation^16^ and Sundarban extent and braided region determined from satellite imagery.

Here, we will be focusing on the Ganges Brahmaputra Meghna Delta (GBMD) (Fig. 1), a large and complex delta comprising much of Bangladesh and coastal West Bengal (India), that exhibits many of the aforementioned disturbances and complications. The GBMD is over 100,000 $\text{km}^{2}$ in area. The channels of the GBMD have a wide range of scales, with channels as wide as 4 km and channels as narrow as a few meters. The three widest rivers, that provide the main fluvial input of the GBMD, discharge incredibly large amounts of water and sediment (close to $1.7\times 10^{5}$ cubic meters of water per second and $10^{6}$ tons of suspended sediment per day during flood events^13^). The GBMD experiences large fluctuations in this rainfall and sediment load due to its proximity to the Himalayan mountain range and the southwest monsoons^14^. While some deltas are predominantly tidal, fluvial, or wave dominated^15^, the GBMD has areas that are tidally dominated, areas that are fluvially dominated, and areas that are considered inactive as they are too far from the coast to be influenced by tides and too disconnected from the three main rivers to be significantly fluvially influenced (Fig. 1). The tidally active section of the delta contains a nationally protected mangrove forest called the Sundarbans, that has for the most part been undisturbed by human modification. Directly adjacent to the Sundarban forest is a region of anthropogenically modified land parcels that have been poldered (embanked on all sides) to allow for agriculture and aquaculture development and provide some protection from seasonal flooding^16^ (Fig. 1).

The GBMD is experiencing changes to its tidal extent which is causing seasonal groundwater salinity intrusion^17–21^. Extensive embankments and changes to land use have caused increased rates of subsidence that lower the affected region’s ability to combat sea level rise^16,22–24^. The natural flow of water and sediment in many internal channels has been altered using dam construction and sediment dredging to better suit navigational pathways and water delivery to inhabited areas^25–27^. In addition to the changes that have already occurred in the delta, there are future plans underway by the neighboring Indian government to divert water and sediment from the main Ganges and Brahmaputra Rivers to water scarce regions of India before they enter the delta network^28,29^ which would in turn affect sediment aggradation rates^30^. All of these factors have caused the GBMD to be under increasing threat and thus, an area where identifying hotspots of morphological change is necessary. As this delta is one of the largest and most complex systems in the world, the analysis methods that we develop here can be used in the analysis of other river deltas.

In this study, we analyze thirty years of satellite imagery (1989 to 2019) of the GBMD in order to identify hotspots of naturally and anthropogenically caused changes to the channel system as a whole. Channel networks are extracted from the imagery each year using two different methods. First, we use RivaMap, a method that has been proven to extract channel presence accurately across multiple channel scales^31,32^. When using RivaMap, channel presence is detected in a non-binary manner and channel strength can be affected by channel bank movement, channel centerline movement, depth changes, and changes to the suspended sediment load inside the channel. We also extract channel presence from imagery using DeepWaterMap, a fully convolutional neural network trained to distinguish water from land, snow, ice, clouds, and shadows^33,34^. DeepWaterMap extracted channel imagery represents a nearly binary interpretation of channel presence where near bank water and channel centerline water are equally represented. While both imagery extraction methods allow us to observe real physical changes to the channel network, the RivaMap imagery analysis allows us to quickly identify the regions where the most overall change is occurring in the delta regardless of the mechanism of change, while the DeepWaterMap imagery analysis allows us to single out and quantify the impact of channel bank movement. The extracted RivaMap and DeepWaterMap channel systems are then used to compute two Channelized Response Variance (CRV) maps. The CRV is a metric we developed to track channel morphodynamics and quantify the spatial and temporal distribution of channel change in delta systems through time^35^.

**Figure 2:**
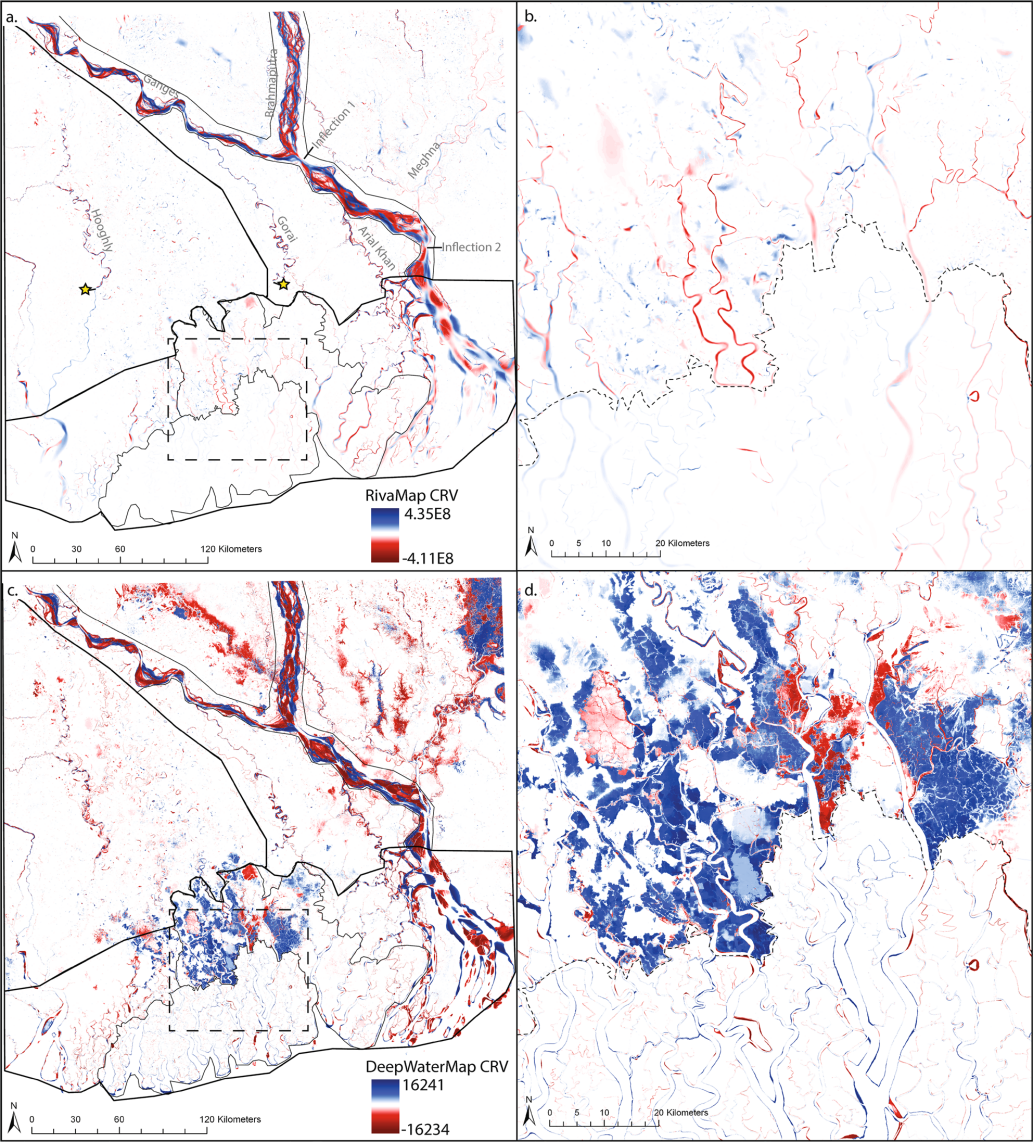
CRV analyses of the GBMD from 1989 to 2019 with zone outlines from Fig. 1 overlain. Blue colors indicate increases in the channel presence and red colors indicate decreases in the channel presence. Intensity of color represents magnitude of CRV. (**a**) RivaMap CRV results for the entire delta with yellow stars to indicate where CRV decrease occurs in Hooghly and Gorai Rivers. (**b**) RivaMap CRV zoomed view of border (dashed line) between the Sundarbans and the Polders outlined with dashed square in (**a**). RivaMap results were created using RivaMap (https://github.com/isikdogan/rivamap) and CRV analysis (https://github.com/passaH2O/CRV-Analysis). (**c**) DeepWaterMap CRV results for the entire delta and for the (**d**) zoomed border between the Sundarbans and Polders. DeepWaterMap results were created using DeepWaterMap (https://github.com/isikdogan/deepwatermap) and CRV analysis.

The RivaMap CRV (Fig. 2a,b) and the DeepWaterMap CRV (Fig. 2c,d) of the entire delta extents are compared to the spatial distribution of a variety of known physiographic zones (Fig. 1) in order to ascertain what is causing hotspots of morphological change in the network and what the signature of geomorphic change is in each zone. Together, these methods allow us to observe a large and complex system and observe how the presence of channels varies through space and time from 1989 to 2019.

## Results

### Detecting hotspots of morphological change by zone

**Figure 3:**
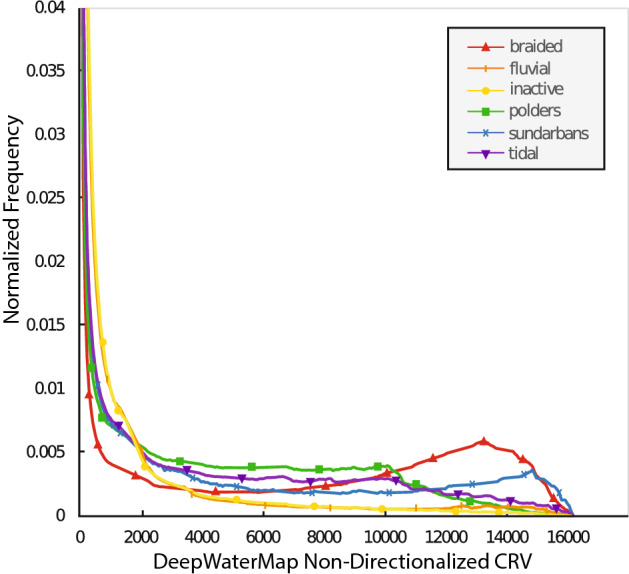
Normalized frequency distributions for each physiographic zone of the GBMD.

By observing the RivaMap CRV and the DeepWaterMap CRV, we can see that in both analysis techniques, the most channel change (highest CRV magnitude) is occurring in the braided fluvially active zone of the delta (Fig. 2a,c). By looking at the normalized distribution of the magnitudes of DeepWaterMap CRV values in the braided zone, we can see that when compared to other regions of the delta, this zone has the lowest frequency of low CRV magnitude pixels and the largest frequency of high CRV magnitude pixels (Fig. 3). This observation of high morphological change in the braided zone is also reflected in the fact that this zone has the largest mean value of non-directionalized DeepWaterMap CRV (Table 1). The mean value of directionalized DeepWaterMap CRV is negative, indicating that the net result of channel bank movement has been a decrease in the channel presence in the braided zone (Table 1). We also observe that the Meghna River is much less active (lower CRV magnitudes) than the Brahmaputra and Ganges Rivers because unlike the Ganges and Brahmaputra, the Meghna is relatively single thread. We are able to identify two inflection points of stability along the braided active extent that have both low magnitudes of geomorphic change and small spatial spread of geomorphic change compared to the rest of the braided zone (Fig. 2a,b).

**Table 1:** Summary statistics of DeepWaterMap CRV by physiographic zone.

Zone	Mean value of directionalized CRV	Mean value of non-directionalized CRV
Sundarbans	1,892.4	5,311.2
Polders	3,146.3	4,722.4
Braided	− 972.7	7,428.3
Fluvial	− 886.9	1,969.1
Inactive	− 417.9	1,594.8
Tidal	1,621.0	4,467.6

There are two main rivers in the non-braided fluvially active region: the Gorai and the Arial Kahn (Fig. 2a). By observing both the RivaMap CRV and DeepWaterMap CRV (Fig. 2a,c) of the Gorai, we can see that the northern extent of the Gorai displays patterns of a meandering river, but the largest patterns of geomorphic change are observed in the middle section. Here there are similar patterns of channel meandering, but the area experiencing geomorphic change is much larger than northward. In the southern extent of the Gorai, there is a point where the levels of morphological change drastically drop (see star, Fig. 2a,c). This point is nearly coincident with the mapped border between the fluvially active and tidally active zones of the delta. The Arial Khan has similar magnitudes of morphological change as the Gorai, but displays a spatial distribution consistent with a highly meandering system through its entire extent rather than just in the middle section.

The inactive zone of the delta displays very low levels of morphological change for much of its extent. The one exception to this pattern is the middle portion of the Hooghly where there are hotspots of morphological change we can observe in both the RivaMap CRV and the DeepWaterMap CRV (Fig. 2a,c). Similarly to the fluvial non-braided zone, this channel displays deposition and erosion patterns consistent with a highly meandering system. The magnitude of morphological change in the Hooghly then drastically reduces southward (see star, Fig. 2a,c). This change happens at a similar latitude as where the Gorai experiences its reduction in magnitude and spatial distribution of geomorphic change. By comparing the normalized frequency distribution of the non-braided fluvial zone and the inactive zone (Fig. 3), it can be seen that these two zones have remarkably similar patterns of morphological change. The one difference between the two distributions is that the non-braided fluvial zone has a slightly higher frequency of the largest magnitudes of DeepWaterMap CRV. This difference can also be observed by comparing the mean values of non-directionalized DeepWaterMap CRV (Table 1). Additionally, like the braided zone, the non-braided fluvial zone and the inactive zone have negative mean values of directionalized DeepWaterMap CRV, indicating that these zones too have been characterized by a net decrease in channel presence (Table 1).

Looking at the tidal zone as it compares to the fluvial dominated zones, we can see that the active channels of the fluvial zones (the braided channels, the Gorai, and the Arial Khan) appear to have larger magnitudes of change than their tidal channel counterparts in both the RivaMap CRV and the DeepWaterMap CRV. The normalized frequency distribution of the tidal zone DeepWaterMap CRV (Fig. 3) reveals that the tidal zone does have a much higher frequency of low CRV values and lower frequency of high CRV values compared to the braided zone. This pattern is also reflected when comparing the non-directionalized DeepWaterMap mean CRV values for each zone (Table 1). However, when comparing the frequency distribution of the tidal zone and the fluvial non-braided zone, we see that although the former does have a higher frequency of low CRV values than the latter, the tidal zone actually has a higher frequency of high CRV values when compared to the fluvial non-braided zone. By looking at the DeepWaterMap CRV we can see that these high CRV values come from the fact that although there is less channel mobility in the tidal zone, there is a large amount of coastal erosion occurring that produces these high CRV values. This causes the mean value of non-directionalized CRV in the tidal zone to be larger than in the fluvial non-braided zone (Table 1). The directionalized mean CRV value of the tidal zone as a whole is positive, indicating that in this region there is a net increase in channel presence (Table 1).

Looking at a zoomed subsection of the delta depicting an area on the border between the natural Sundarban forest and the anthropogenically modified poldered region directly north (Fig. 2b, d), it can be seen that there is a clear divide in the magnitude of the CRV in both the RivaMap CRV and the DeepWaterMap CRV. This difference between the Sundarbans and the embanked Polders is also apparent when comparing the normalized frequency distributions of the two zones (Fig. 3). At very low CRV magnitudes, both the poldered zone and the Sundarbans have similar frequencies. At the mid-level CRV magnitudes, the poldered zone has much higher frequency of occurrence than the Sundarban zones. At the highest CRV magnitudes, the Sundarbans actually have a higher frequency of occurrence than the poldered zone. These patterns are also reflected in the mean non-directionalized CRV values where the Sundarbans have a slightly higher mean CRV than the Polders even though as a whole it appears as though the Sundarbans have lower magnitudes of CRV (Table 1). These patterns are discussed more extensively in the following sections. Similarly to the entire tidal zone, the Sundarbans and the Polders both have positive mean directionalized CRV values (Table 1), meaning they too are characterized by net increase in channel presence.

### Centerline analysis

**Figure 4:**
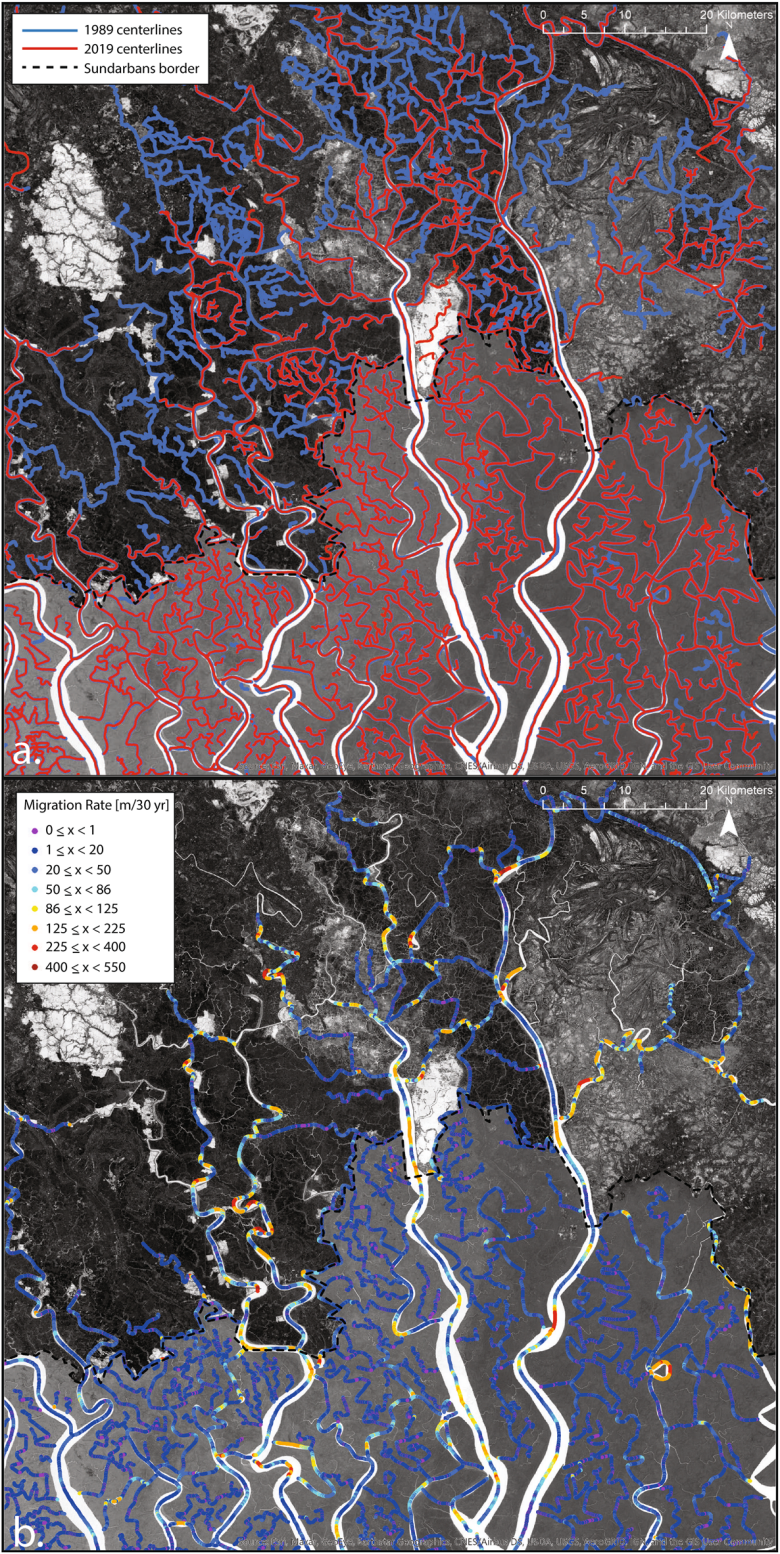
Centerline analysis for the zoomed view (dashed square from Fig. 2) of the border between the Sundarbans (south) and the Polders (north). Underlying imagery created by calculating the MNDWI^38^ using Landsat imagery mosaicked from all cloud free images from 10/1/1989 to 3/31/1990. (**a**) Centerlines for 1989 shown in blue and centerlines for 2019 shown in red. (**b**) Migration rates of the subsection of centerlines from 1989 to 2019.

In order to gain more information about the cause of the CRV discrepancy we observe between the Sundarbans and the Polders, centerlines from 1989 and 2019 are extracted from RivaMap imagery^31,32^ and compared to one another (Fig. 4a). Although there are a few areas of the Sundarbans that have infilled or have newly incised channels, for the most part, the Sundarbans channel centerlines are stable from 1989 to 2019. The Polders contrastingly have a large majority of the smaller tidal channels being infilled from 1989 to 2019.

**Figure 5:**
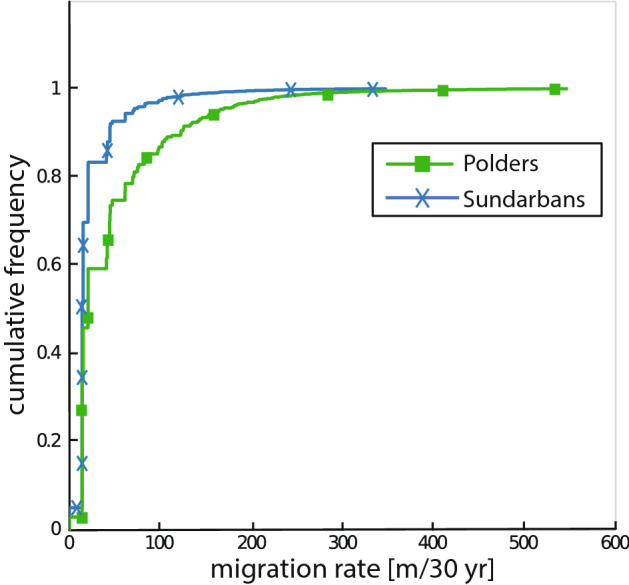
Cumulative frequency distributions of migration rates for the Polders and the Sundarbans.

To compare channel centerline migration rates, every channel that appeared in both 1989 and 2019 that was larger than 60 m wide was isolated from the full centerline network (Fig. 4b). Since these channels persisted from 1989 to 2019, migration rates could be determined. The migration rates as computed represent the total distance migrated over the course of the entire 30 year time span (Fig. 4b). We find that the average channel migration rate in the Polder zone is 46.8 m per 30 years with a maximum of 546.0 m per 30 years and the average channel migration rate in the Sundarban zone is 31.0 m per 30 years with a maximum of 347.3 m per 30 years. Additionally, by plotting the cumulative distribution function for each of the sets of migration rates (Fig. 5), it can be seen, for example, that 25% of the poldered channels have migration rates that exceed 60 m per 30 years, while only 6% of the Sundarbans channels have migration rates that exceed 60 m per 30 years. Finally, a non-parametric Wilcoxon rank sum test was conducted using both sets of migration rates and it was found that the null hypothesis (stating that the data points come from the same distribution) was rejected (*p* value = $5\times 10^{-7}$). Together these analyses demonstrate that the migration rates of the poldered zone channels and the Sundarban channels are significantly different from one another.

## Discussion

### Fluvially active versus tidally active zones

Our results suggest that if coastal erosion is not considered, fluvial processes (braiding and meandering) cause larger magnitudes of geomorphic change than tidal processes. This is consistent with other studies that have looked at the mobility of tidally influenced versus fluvially influenced channels^39–41^. We find that the braided fluvially active section has the largest magnitudes of geomorphic change. This is because in these sections, channels can drastically change their presence from being filled with water to being abandoned, as the distribution of water and sediment are routed along different abandoned paths.

Again excluding the effects of coastal erosion, we find that the Hooghly, Gorai, and Arial Khan rivers also have higher magnitudes of geomorphic change than the tidally active section, but do not have magnitudes of change quite as high as the braided fluvially active section. This relative lower magnitude of change is because in the non-braided fluvially active zone, the main form of geomorphic change is channel meandering. While channel braiding causes change in channel presence along nearly the entire width of the multi-thread braiding area, channel meandering only causes changes in channel presence along the inner depositional and outer erosional banks of the curved sections of the channel. The two inflection points of low geomorphic change along the braided section, as well as the Meghna River low geomorphic change magnitudes, indicate that those areas could be considered more stable than the rest of the braided zone and could be more suitable for permanent urban development.

We also find that as a whole, the non-braided fluvial zone and the inactive zone have nearly identical normalized frequency distributions (Fig. 3). This result suggests that segmenting the delta as being fully inactive in the western inland area and fully active in the eastern inland area may not be the best way to represent the delta as it evolves through time. Instead, it may be more appropriate to label the extents of Hooghly, Gorai, and Arial Khan as fluvially active zones, and the remainder of the non-braided areas inland as inactive zones.

This hotspot analysis of the entire extent of the GBMD also allows us to discern the inland extent of tidal influence on changes in channel morphology in the 30 years of observation. In the lower section of both the Gorai and the Hooghly Rivers there are points along the same latitude (23.02° North) where the magnitude of morphological change drastically drops (Fig. 2a). These points may represents where the dominant influence on channel morphology and mobility shifts from fluvial to tidal. There is also a slope break of the delta near this extent that could also play a role in the sudden change of channel stability at this latitude^42^. The extent of saline groundwater^43,44^ and the distance upstream that tidal influence can be felt in the water level have been analyzed for the GBMD^24,45^, but with this analysis, we can now understand where those extents have significant effect on channel movement and have a framework to examine how that extent evolves through time.

Finally, looking at the mean values of the directionalized DeepWaterMap CRV analysis for each zone (Table 1), we can see that the three zones subject to tides (Tidal, Sundarbans, and Polders) all have positive net values of DeepWaterMap CRV, while the three inland fluvial zones (Fluvial, Braided, and Inactive) all have net negative values of DeepWaterMap CRV. This suggests that in the last 30 years, more land area has been gained in the inland fluvial zones and more land area has been lost in the more coastal tidal zones. This loss of land area in the tidal zone is partially due to the coastal erosion occurring as well as the appearance of the shrimp ponds.

### Polders versus Sundarbans zones

The results of the analysis border between the Polders and the Sundarbans suggest that the Polders are experiencing a much larger magnitude of geomorphic change than the Sundarbans are. It can be seen that many channels immediately change their magnitude of RivaMap CRV upon crossing the border between the two regions (Fig. 2b). After validating these results by checking the original multi-spectral imagery, we suspect that this result is less due to changes in bank location and more a result of the appearance of multiple non-channelized water bodies directly adjacent to the poldered channels. These positive zones of RivaMap CRV are the result of the appearance of shrimp ponds within the Polders^16^. The steady decline of freshwater inflow over time from the Gorai^46^ has caused increases in dry-season surface water salinity that makes the soil less fertile for traditional rice farming. This process, in addition to government policies making shrimp farming economically preferable, has caused many farmers to change from rice farming to shrimp farming. Shrimp ponds have exposed water surface at all times, whereas rice farms have sprouts growing out of the water surface causing it to have more of a land signature in the imagery. This change from rice to shrimp farming has caused the creation of the positive zones of change we observe in the RivaMap CRV. The ponds’ direct adjacency to the poldered channels causes the RivaMap method to detect the entire channel and shrimp pond as a single extra wide channel feature with a lower channel strength than a channel alone with no adjacent pond feature. Therefore, the appearance of the ponds causes some of the RivaMap poldered channels to appear as if they are experiencing large decreases in channel intensity, when in fact that may not be the case.

Because the RivaMap CRV is affected by the presence of pond features not present in the Sundarbans, to compare the two zones, we focus on the DeepWaterMap CRV results. Looking at the zoomed DeepWaterMap CRV results (Fig. 2d), it can be seen that there are still large and clear differences between the Polders and the Sundarbans. The comparison of the normalized frequency distributions of the two zones (Fig. 3) shows that although the Polders have higher frequency of mid-level CRV magnitudes, the Sundarbans zone has higher frequency of the lowest and the highest CRV magnitudes. The Sundarbans high frequency of low CRV magnitudes matches the patterns seen in the DeepWaterMap CRV (Fig. 2d): many channels show very little change occurring along their edges. The higher frequency of the highest CRV values seems counter intuitive at first especially when looking at the DeepWaterMap CRV map that appears to show much less change occurring in the Sundarbans (Fig. 2d). However, as previously noted, much of the CRV in the poldered zone is actually due to the appearance of shrimp ponds directly adjacent to the channels. While the DeepWaterMap distinction between channels and land is binary, these shrimp pond zones have values above zero but below the maximum value of 255 that is assigned to channels. Thus, the CRV values of these appearing pond features have lower magnitudes than the changes in the channels themselves. This result, combined with the fact that the Sundarban region is experiencing coastal erosion while the Polders are inland, is what causes the Sundarbans to have higher frequency of the highest CRV values.

While the appearance of the shrimp ponds is an interesting anthropogenic change to detect in the CRV analyses, this change is more just a simple land-use change rather than true geomorphic change. In order to assess the effect of the anthropogenic modifications on just the channelized areas and not the shrimp ponds, we look to the channel centerline migration analysis results. The migration analysis shows that there was extensive infilling in the poldered area compared to the Sundarbans (Fig. 4a) and that there was a statistically significant difference in the migration rates of the channels in the two zones (Figs. 4b, 5). This higher magnitude of morphological change to the centerlines in the Polders may be due to the large degree of anthropogenic modification that has gone into the poldered region to make it inhabitable and able to be farmed. The embankments that characterize the poldered land cut off smaller tidal channels that are no longer connected to the main channel system^16^. The disappearance of many of these smaller tidal channels is more of a land use change (physically cutting off the channels from water sources causing infilling) rather than a true geomorphic response. Additional infilling of the channels around, but not contained in, the embanked land, however, can be considered a true geomorphic change. This type of infilling is due to the local reduction in the tidal prism caused by Polders^47^. A reduced tidal prism causes corresponding reductions to the channel water velocities which cause increases in sediment deposition. This sediment deposition can reduce the depth and width of channels and eventually infill channels completely^48,49^. This increased sediment deposition could be partially responsible for the increase in the magnitude of morphological change as the channels cross from the Sundarbans to the Polders.

Although there are many channels that are infilling, our migration analysis also showed that some channels in both the Polders and the Sundarbans are laterally migrating and meandering. The channel migration analysis conducted shows that migration rates of channels in the poldered zone are higher than migration rates in the Sundarbans (Fig. 4b). This increase in channel migration and meandering could be a response to the perturbations in the anthropogenically modified areas. It has been shown that tidal channels can undergo widespread reorganization in response to changes in water and sediment flow^48,50^. By causing infilling and changing the tidal prism, the Polders have caused perturbations that could have triggered channel reorganization events in several tidal channels that, in turn, cause increased channel migration rates in the Polders relative to the Sundarbans.

## Conclusions

The Ganges Brahmaputra Meghna Delta (GBMD) is the largest and most populated river delta in the world and as such, it is critically important to characterize the morphological changes that are occurring to the channel network. Using the Channelized Response Variance (CRV) method to track the variance in channelization through time, we have conducted a channel change analysis on the entire extent of the GBMD and quantified relative magnitudes and directions of morphological changes occurring in the channel network. By looking at the entire extent of the delta at the same time, we are able to see how known zones of different forcings (Fig. 1) are affecting the system relative to one another. We found that the fluvial zone is the most active with the highest values of morphological change (Fig. 2a,c). Within the fluvially active zone, the braided sections of channels are the most active while the active meandering channels are less active (Fig. 2a,c). We found that there was a clear decrease in CRV as channels leave the fluvially active zone and enter the tidally active zone (Fig. 2a,c). The location of this decrease represents a shift from fluvial influence dominance to tidal influence dominance on the channel morphology. Finally, we also found a distinct decrease in magnitudes of geomorphic change as channels leave the embanked poldered zone and enter the Sundarban forest (Fig. 2b, d). The cause of this change is primarily channel infilling within the Polders, around the Polders due to a reduction of the tidal prism, and tidal channel reorganization causing channel migration in response to perturbations to the system by anthropogenic change (Figs. 2d, 4a, b). Many of the results found in this paper are consistent with the findings of other recent studies in this area^16,48,48,51^, showing that there is convergence between remote-based and field-based analysis approaches.

These results suggest that anthropogenic modifications have been the cause of significant changes to the channel network of the GBMD. While some changes in delta networks occur over millennial timescales, the changes we observe here are occurring over decadal timescales. The fact that the adjustments to this highly populated delta are occurring over human lifespan timescales makes it especially important to address the impact of human activity on river deltas and use this information to plan future necessary modifications to the GBMD.

## Methods

Images encompassing the entire extent of the GBMD are acquired from Landsat 5 TM, Landsat 7 ETM+, or Landsat 8 OLI, depending on availability. Images are chosen during the dry season (October–March) where cloud cover is lower, and channels are consistently at their lowest stage. For each year a single composite image is created by taking the average value of all cloud free pixels during the dry time period. By taking only images from the dry season and averaging every value, we are able to reasonably constrain the effects of changes in stage height. This method allows us to obtain very clear and continuous images and also dampens unavoidable fluctuations in tidal levels. We find that the averaged tidal level (collected at Hiron Point) is very similar for the four partially overlapping Landsat paths that cover the study area ($164.2 \pm 17.3\,\text{cm}$). We find that there is no significant increasing or decreasing trend to the tidal level through time for each path. Because of these findings, we proceed assuming that any change over time we observe in the imagery will be due to geomorphic change and not tidal fluctuations.

Once a single composite image for each year has been acquired, a grayscale water emphasized image is created by calculating the Modified Normalized Difference Water Index (MNDWI)^38^. The MNDWI uses a combination of the green and SWIR bands to enhance open water features and suppress the soil and vegetation classes. The MNDWI images are then analyzed with RivaMap, a method for automatic extraction of channel networks from remotely sensed imagery^31,32^. RivaMap extracts non-binary channel presence across a large range of scales found in many river delta channels by computing a multi-scale singularity index (SI) response on the MNDWI intensity image^31,52^. The SI response images represent where channelized water is present and how strong the intensity of that channel is. Channel intensity can be affected by water depth and sediment content, so the SI value at the center and edges of channels is often different. Additionally, if bank erosion is small compared to the changes occurring in the center of the channel, the SI can occasionally miss bank geomorphic changes. While under some circumstances having a non-binary representation of channel presence gives us useful information about overall change occurring, it is also insightful to have a representation of channel presence where the middle and edges of the channel are represented equally, allowing for the analysis of just the effect of channel bank movement. For this reason, we produce a second set of images using DeepWaterMap, a fully convolutional neural network that has been trained to extract water features from land, snow, ice, clouds, and shadows^33,34^. We observe that DeepWaterMap produces water presence quasi-probability maps where after normalization, land pixels are given a value of 0, channel features are given a value of 255, and non-channelized land features (such as the shrimp ponds and flooded poldered areas) are given values in between, effectively resulting in an almost binary channel map.

We then compute the Channelized Response Variance (CRV) on both the RivaMap imagery set of 30 images and the DeepWaterMap set of 30 images^35^. The CRV is a method that has been shown to quickly and accurately quantify temporal and spatial trends in channel variation from imagery by tracking changes to the channelized response through time^35^. To compute the CRV image, the RivaMap and DeepWaterMap images for each year are arranged in a time-ordered 3-D array and the variance is calculated for each pixel in the time dimension. Higher values of CRV (hotspots) indicate that the pixel experienced larger differences between being identified as a channel and being identified as land. Smaller values of CRV indicate that the pixel did not experience significant change between being a channel and being land. To add directionality to the magnitude of CRV, we conduct a linear regression analysis on each pixel’s time series of channel response. Although the time series may not always exhibit a linear trend, the slope of the linear regression analysis returns accurate information on whether the CRV observed is due to an increase or a decrease in channel presence^35^. In the DeepWaterMap analysis, the maximum CRV occurs when a pixel is represented as land (a value of 0) for half of the time and represented as a channel (a value of 255) for the other half of the time. Therefore, a pixel that is experiencing unidirectional change and changes from land to channel in the middle of the time frame of analysis will return the highest CRV. A pixel that experiences unidirectional change that changes from land to water near the beginning or end of the time frame will return a smaller CRV value because it remained stable for more than half of the time. A pixel experiencing non-monotonic change between being a channel and being land would return a high CRV, but these areas are filtered out of the analysis by using the linear regression data. Points with non-monotonic trends in channel presence will return slope values of zero. Points with high CRV and zero slope values have their CRV multiplied by zero to remove them from the CRV analysis and allow us to only focus on pixels experiencing monotonic change. For a more detailed explanation of what CRV signature to expect from different channel movements, see the Supplementary Table S1 provided in Jarriel et al.^35^.

The DeepWaterMap CRV of the entire GBMD is then divided into sub-sections based on physiographic zones (Fig. 1). The magnitude of DeepWaterMap CRV values for each zone are used to create a normalized frequency distribution curve for each zone (Fig. 3). This operation allows us to compare the DeepWaterMap CRV magnitude distribution for each zone and begin to ascertain what may have caused the differences we observe.

Centerlines are extracted via non-maxima suppression of the SI response along the direction of the channel. To conduct the centerline comparison, centerlines from 1989 and 2019 are separated from the full set and overlaid on top of one another. Channels that persist from 1989 to 2019 that are greater than 60 m wide are used in the migration rate analysis. Migration rate of the channels is determined using the ArcGIS ‘near’ function. Migration rates for the poldered zone and the Sundarbans zone are separated and a cumulative distribution function for each zone is created.
